# A survey of transcutaneous blood gas monitoring among European neonatal intensive care units

**DOI:** 10.1186/1471-2431-5-30

**Published:** 2005-08-10

**Authors:** Mario Rüdiger, Kerstin Töpfer, Hannes Hammer, Gerd Schmalisch, Roland R Wauer

**Affiliations:** 1 Clinic of Neonatology; Universitätsmedizin Berlin, Charité-Mitte; 10098 Berlin; Germany; 2 Department for Neonatology, Medical University Innsbruck, Department for Neonatology, 6020 Innsbruck, Austria

## Abstract

**Background:**

PCO_2 _and PO_2 _are important monitoring parameters in neonatal intensive care units (NICU). Compared to conventional blood gas measurements that cause significant blood loss in preterms, transcutaneous (tc) measurements allow continuous, non-invasive monitoring of blood gas levels. The aim of the study was to survey the usage and opinions among German speaking NICUs concerning tc blood gas monitoring.

**Methods:**

A questionnaire was developed and sent to 56 head nurses of different NICUs in Germany, Switzerland and Austria.

**Results:**

A completely answered questionnaire was obtained from 41 NICUs. In two of these units tc measurements are not performed. In most NICUs (77%), both P_tc_O_2 _and P_tc_CO_2 _are measured simultaneously. Most units change the sensors every 3 hours; however, the recommended temperature of 44°C is used in only 15% of units. In only 8% of units are arterial blood gases obtained to validate tc values. Large variations were found concerning the targeted level of oxygen saturation [median upper limit: 95% (range 80–100%); median lower limit: 86% (range 75–93%)] and PO_2 _[median upper limit: 70 mmHg (range 45–90 mmHg); median lower limit: 44 mmHg (range 30–60 mmHg)].

**Conclusion:**

Our survey shows that the use of tc monitors remains widespread among German speaking NICUs, despite earlier data suggesting that their use had been abandoned in many NICUs worldwide. In addition, we suggest that the current method of monitoring oxygenation may not prevent hyperoxemia in preterm infants.

## Background

Preterm infants are vulnerable to alterations in arterial oxygen or carbon dioxide tension [1]. Changes in oxygen supply contribute to the subsequent development of retinopathy of prematurity or bronchopulmonary dysplasia [2]. Hypocarbia has been associated with the subsequent development of periventricular leucomalacia [3] and cerebral palsy [4], and while hypercarbia may protect the perinatal brain from hypoxemic-ischemic damage [5,6], it could also cause retardation of retinal vascularization [7].

Despite an ongoing discussion concerning the optimal values of blood gas levels, there is consensus that the partial pressures of arterial oxygen and carbon dioxide (P_a_O_2 _and P_a_CO_2_) should be kept within a narrow range. Thus, intermittent or continuous determination of blood gases is required. However, the repeated arterial blood sampling that is required for the correct measurement of P_a_O_2 _and P_a_CO_2 _is difficult to perform in preterm infants because the usage of indwelling catheters is associated with complications and significant blood loss. Capillary blood samples, which are painful but easier to obtain, provide satisfactory values for P_a_CO_2 _but tend to underestimate P_a_O_2 _[8].

Transcutaneous (tc) measurement of oxygen (P_tc_O_2_) and carbon dioxide (P_tc_CO_2_) tension is a non-invasive method that has recently offered some promise [1]. Several studies have shown a good correlation between tc and arterial values [9-11]. However, during routine clinical treatment, several problems – such as burns – appear [1]. Furthermore, a poor correlation between P_a_O_2 _and P_tc_O_2 _was found under routine clinical conditions [12]. On the basis of these reports, alert letters on the subject of tc PO_2 _measurements were published by Canadian and British health authorities [13,14], and after clinical introduction of pulse oximetry, the interest in tc oxygen monitoring decreased was abandoned altogether in many neonatal intensive care units (NICUs) around the world [15]. However, the actual status of tc blood gas monitoring in German speaking NICUs remains unknown.

The present observational study was performed to answer the following questions:

1.) To what extent is tc blood gas monitoring performed in German speaking NICUs?

2.) Given reports that nurses are reluctant to perform tc monitoring because they question its reliability [15], what are the opinions of nurses concerning the reliability of tc values?

3.) Are there any differences between NICUs concerning technical aspects of tc sensor application?

4.) What methods are used to detect hypo- or hyperoxia and what are the upper and lower limits for oxygen saturation and partial pressure in different NICUs?

## Methods

The questionnaire consisted of four main parts and is described below. A pre-test of the questionnaire was performed at the authors' institution. Twenty nurses were asked to answer the questions. Four of the questions in the original version were found to be misleading and were reworded for the final version of the questionnaire.

The final questionnaire was sent to NICUs by ordinary mail. To avoid any bias due to differences in national medical regulations, the questionnaire was distributed only in German speaking countries. From a list of 168 university hospitals in Germany, Austria and Switzerland every third unit was chosen (n = 56 units). Because nurses are mainly responsible for the usage of tc equipment, head nurses were asked to answer the questionnaire according to their institutional guidelines.

### Usage of tc measurements

The first part of the questionnaire was designed to obtain information concerning the usage of tc measurements. The following questions were included:

1. What aged patients do you mainly care for (only preterm infants / preterms and neonates / neonates and older infants)?

2. On which patients do you perform tc measurements (conventional mechanically ventilated patients / CPAP patients / only supplemental oxygen patients / every patient)?

3. Which parameters do you measure (tc PO_2 _/ tc PCO_2 _/ both)?

4. What manufacturer does the monitoring system come from?

### Nursing practice

The second part of the questionnaire consisted of questions concerning nursing practice during tc measurements:

1. How often do you change the site of the sensor (every 1 / 2 / 3 hours / more / less frequently)?

2. Do you think the changes violate "minimal handling" practices?

3. Do you use a special treatment for erythematous skin areas?

### Technical details of tc monitoring

The third section investigated technical details of tc usage and included the following questions:

1. How often do you change the sensor site?

2. What is the temperature of the sensor?

### Accuracy of transcutaneous measurements

The final part of the questionnaire was dedicated to the correlation of tc and invasive blood gas measurements. The following questions were asked:

1. What is your impression concerning the accuracy of tc measurements (good / moderate / poor)?

2. On average, how often do you compare tc values with blood gases (routinely / depending on the values)?

3. What source of blood do you use for validation (capillary / arterial / venous)?

4. Concerning monitoring of oxygenation in preterm infants, which value is more important when estimating hypoxia (saturation / tc PO_2_) and hyperoxia (saturation / tc PO_2_)? What are the lower and upper limit values?

### Statistics

Data were analyzed with descriptive statistics using Excel (Microsoft) software. Data are presented as median and range or as relative percentages where appropriate.

## Results

The questionnaire was completed by 41 of the 56 NICUs (73%). Among the 41 units with completed questionnaires, 2 did not perform tc measurements and hence were excluded from the subsequent analysis.

The head nurses of the 15 non-responding units were contacted by telephone by which it was confirmed that no tc measurements were performed in 8 units, tc measurements were performed but no further information was voluntarily offered in 4 units, and no information at all was offered in 3 units.

### Usage of tc measurements

**Figure 1:**
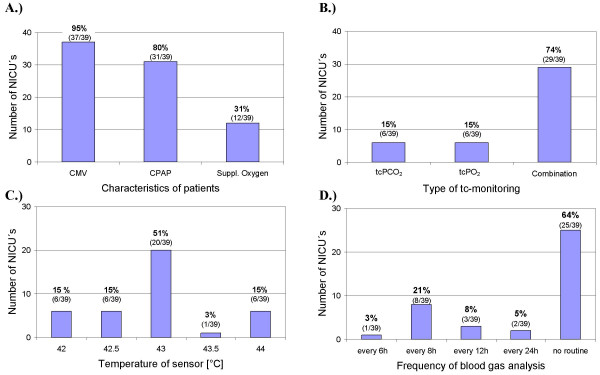
Shown are the number of NICUs. (A) that use tc monitoring on patients on conventional mechanical ventilation (CMV), continuous positive airway pressure support (CPAP) or supplemental oxygen (suppl. oxygen) [multiple answers were possible]; (B) that use a combination of tc PO_2 _and tc PCO_2 _sensors (Combination), or a single sensor; (C) that use a sensor temperature of 42°, 42.5°, 43°, 43.5° or 44°C; (D) that compare tc values with blood gases routinely every 6, 8, 12 or 24 h or do not have a specified routine.

Most of the evaluated NICUs (28/39) mainly care for preterm and term new-borns. In the remaining 11 NICUs, both new-borns and infants are treated. Most of the units perform tc measurements on mechanically ventilated infants or on infants on continuous positive pressure (CPAP) support (Fig. 1A). About 30% of units also use tc monitoring for infants on supplemental oxygen.

The majority of the answering units use a combination of tc PO_2 _and tc PCO_2 _sensors. Some units use either tc PO_2 _or tc PCO_2 _and two units use both sensors separately (Fig. 1B).

Devices for tc blood gas measurements were from multiple suppliers; however, Radiometer was the most commonly used manufacturer, followed by Hewlett Packard and Hellige.

### Nursing practice

Analysis of handling showed that most units change the site of the sensor every 3 hours or even more frequently, 6 of 39 units change the sensor every 4 hours, and 3 of 39 less often than every 4 hours. There was no correlation between frequency of changes and manufacturer.

In 60% of the participating units, nurses considered the changing of the sensor as a discomfort for the patient and a violation of the minimal handling policy.

About one third of the units do not have a special treatment of erythematous sensor areas, whereas the remaining units use various ointment therapies.

### Technical details of tc measurements

Large differences were found concerning the technical aspects of tc blood gas monitoring. In 17 of 39 units, sensors are calibrated after each change of sensor site. A routine calibration of the sensor is performed every 4 hours in 8 units and once daily in 11 units.

Sensor temperature mainly depends on the age of the patient; however, in most units the sensor works at a temperature of 43°C. In some units temperatures between 42 and 44°C are used (Fig. 1C).

### Individual opinion concerning the accuracy of transcutaneous values

**Figure 2:**
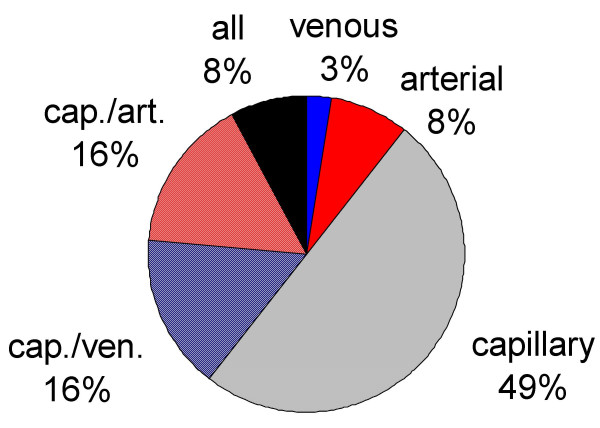
Distribution of blood sample type used to compare tc values.

Invasive blood gas measurements are routinely performed for comparison with tc values in 14 of 39 units (Fig. 1D). Blood gases are mainly obtained from capillary blood, with only 8% of units obtaining arterial samples (Fig. 2).

The majority of respondent nurses considered the tc measurements as a good (29/39) or intermediate (9/39) estimate of arterial blood gases, while only one NICU nurse stated that tc measurements lead to poor estimates.

The question concerning hyperoxia and hypoxia detection was answered by only 35 units, but these provided some interesting data. To detect hyperoxia in preterm infants, 16 of the 35 NICUs use only oxygen saturation, 10 use only P_tc_O_2_, 8 use both and 1 uses neither method. The median upper limit for saturation was 95% (range 80–100%) and the median upper limit for P_tc_O_2 _was 70 mmHg (range 45–90 mmHg).

To detect hypoxia, the majority (24/35) of units use only saturation, whereas 9/35 units use both methods (P_tc_O_2 _and saturation) and two units use only invasive blood gas methods. The median lower limit for saturation was 86% (range 75–93%) and the median lower limit for P_tc_O_2 _was 44 mmHg (range 30–60 mmHg).

## Discussion

To prevent acute or chronic damage, blood gases must be monitored in preterm infants [1]. Transcutaneous (tc) measurement of blood gases represents a valuable tool for continuous, non-invasive monitoring.

Tc monitoring is associated with several problems and it has been reported that this type of monitoring has been abandoned in many NICUs around the world [15,16]. Up until now, no data were available concerning the usage of tc monitors in German speaking neonatal units. In our observational study, we received answers from 41 of 56 NICUs approached. Of these, 39 responded that they currently use tc monitoring. Four of the 15 non-responding units also performed tc measurements but did not provide any further information. Thus, our representative survey suggests that at least 43 of 56 NICUs (77%) use tc blood gas monitoring. In six units tc monitoring had been completely abandoned. In contrast to data that suggest a reluctance of nurses to use tc monitoring [15], our study shows wide acceptance of the technique among NICU nurses. The majority of surveyed nurses stated that the accuracy of the tc readings is mostly reliable. However, the need for frequent changes in sensor sites was considered a violation of the minimal handling policy.

Tc monitoring and pulse oximetry are useful techniques for the non-invasive monitoring of oxygenation in new-borns that require supplemental oxygen. Whereas capillary blood gases and pulsoximetry are sufficient to detect hypoxia, it is not sufficient to use either method to prevent hyperoxia. Nevertheless, in the present survey 16 of 35 NICUs used only saturation to detect hyperoxia. Since pulse oximetry values cannot be used to detect hyperoxia, arterial PO_2 _should also be measured intermittently. About half of all answering units stated that they perform blood gas analysis exclusively from capillary blood samples, but capillary PO_2 _estimations can only exclude hypoxia and are insufficient for detecting hyperoxia [17]. Thus, it could be speculated that the current oxygen-monitoring policy of some units exposes infants requiring supplemental oxygen to a higher risk of subsequent development of oxygen associated damage, such as retinopathy [18].

Large variations were found among the different NICUs with regard to the definition of hypoxia and hyperoxia. The upper limit for oxygen saturation ranged between 80% and 100%, and if tc measurements were used, the upper limit ranged between 45 and 90 mmHg (median 70 mmHg). A similarly wide range was found for the detection of hypoxia with a lower saturation border between 75% and 93% (mean 86%). These differences are substantial and could explain some of the described differences in outcomes of preterm infants [19]. These differences require further investigation and specification. The present data do not allow a differentiation between the target values for infants with supplemental oxygen or those with respiratory support.

The present study included some limitations that are partially associated with the chosen method of obtaining information. First, the study sample is based on the return of completed questionnaires. We achieved a return rate of 73%, which is considered a good result and allows reliable interpretation. Secondly, the questionnaire was not designed to identify an association between the monitoring policy at the institution and the clinical outcome parameters; however, the present study does provide sufficient data to plan an appropriate study protocol to address that question. Finally, the questionnaire was only designed to receive information consistent with institutional guidelines. In some cases, the unique situation of an individual patient could lead to deviations from the general policy. A follow-up study could further specify the use of tc monitoring under different clinical conditions (ventilation, oxygen supply, CPAP) and in different populations (preterm, term infants), and could also include the primary reason for the use of tc monitoring. However, surveys of these factors should be mainly performed among the attending neonatologists.

## Conclusion

The present survey provides valuable data concerning the current situation of routine clinical blood gas monitoring in German speaking NICUs and has produced the following conclusions: 1) Transcutaneous blood gas monitoring is frequently used in neonatal intensive care units; 2) large variations exist concerning the targeted range of oxygen saturation or PO_2_; and 3) in infants requiring supplemental oxygen, the current method of monitoring oxygen may not be sufficient to prevent hyperoxia.

## Competing interests

The author(s) declare that they have no competing interests.

## Authors' contributions

MR developed the study design and drafted the manuscript. KT conceived the study, developed the study design, distributed the questionnaire and collected the data. GS was significantly involved in drafting and revising the article and contributed to analysis and interpretation of data. HH helped to coordinate the study and to develop the questionnaire. RW contributed to the conception and design of the study and the interpretation of data.

## Pre-publication history

The pre-publication history for this paper can be accessed here:


